# Randomised Clinical Trial: Effect of AH Plus and Neosealer Flo on Postoperative Pain and Healing of Periapical Lesions

**DOI:** 10.3390/bioengineering12040376

**Published:** 2025-04-02

**Authors:** Juan Algar, Cristian Docampo-Vázquez, Cristina Rico-Romano, Ana Boquete-Castro, Cristina Obispo-Díaz, Juan Manuel Aragoneses

**Affiliations:** 1 Facultad de Odontología, Universidad Alfonso X el Sabio, Avenida de la Universidad 1, 28691 Villanueva de la Cañada, Spain; cdocavaz@uax.es (C.D.-V.); cromaric@uax.es (C.R.-R.); aboqucas@uax.es (A.B.-C.); cobispo@uax.es (C.O.-D.); jaraglam@uax.es (J.M.A.); 2 Department of Clinic Dentistry, Faculty of Biomedical and Health Sciences, Universidad Europea de Madrid, c/ Tajo S/N, Villaviciosa de Odón, 28670 Madrid, Spain; 3 Department of Dentistry, Universidad Federico Henriquez y Carvajal, Santo Domingo 10106, Dominican Republic

**Keywords:** bioceramic sealer, obturation, root canal, endodontic, pain, treatment, ECA

## Abstract

Apical periodontitis is a common inflammatory condition associated with root canal treatment (RCT) failure. The quality of the three-dimensional root canal seal is critical to the success of the treatment. Bioceramic sealants, such as Neosealer Flo, offer biological advantages such as osteoconduction, biocompatibility and sustained calcium ion release, which may improve apical healing. The aim of this study was to compare AH Plus and Neosealer Flo in terms of postoperative pain, extrusion and periapical healing. A single-blind, randomised clinical trial was conducted with 60 patients divided into AH Plus and Neosealer Flo groups. Post-operative pain was assessed using a visual analogue scale (VAS) at 24 and 48 h and at 7 days. Seal quality and periapical healing were assessed at 6 months using the AAE success criteria by clinical and radiographic evaluation. Neosealer Flo resulted in less postoperative pain at 24 h and 7 days compared to AH Plus. Extrusion did not significantly affect pain or correlate with the type of sealer used. Both materials achieved similar periapical healing rates. Neosealer Flo demonstrated advantages in pain reduction, while both sealants showed comparable efficacy.

## 1. Introduction

Apical periodontitis is one of the most common inflammatory diseases in humans. In Western populations, it is estimated that 41% to 59% of individuals have undergone root canal treatment (RCT) and 24% to 65% suffer from this condition [1]. This pathology is primarily due to persistent chronic infections caused by microorganisms from necrotic pulp tissue, leading to bacterial progression to the periapical tissues and resulting in primary apical periodontitis. It can also develop as a consequence of previous RCT failure, resulting in secondary apical periodontitis [1,2,3,4]. The presence of apical periodontitis is considered a negative prognostic factor for RCT success [5].

A key determinant of RCT success is the achievement of effective three-dimensional obturation that prevents the leakage of toxins and bacterial products, thereby blocking communication between the root canal and periapical tissues. Traditionally, this obturation is achieved by a combination of sealer and gutta-percha to create a hermetic three-dimensional filling [6,7]. In this context, the sealer plays a critical role in this by filling the spaces between the obturation material and the canal walls [8]. Radiographic evaluation remains the standard method for assessing obturation quality, focusing on the presence or absence of voids and verifying that the apical termination of the filling is within 2 mm of the radiographic apex [7].

AH Plus Sealer (Dentsply Sirona, Charlotte, NC, USA) is widely regarded as the reference material among root canal sealers due to its favourable physicochemical properties, biocompatibility and proven antimicrobial activity [8]. However, it has certain drawbacks, such as initial cytotoxicity (especially during setting), difficulty in removal, lack of adhesion to dentinal walls and inability to reinforce dental structures [9]. To overcome these limitations, several strategies have been developed to improve the antimicrobial and adhesive properties of endodontic sealants. These include the incorporation of metallic nanoparticles (such as silver, gold and zinc oxide) and plant-based antimicrobial extracts, which help to reduce microbial persistence and the risk of reinfection [10]. At the same time, advances in dental polymer chemistry have led to the development of acrylate-based sealants that offer improved adhesion to dentin and reduced solubility compared to traditional epoxy-based sealants [11]. Among the most promising alternatives, bioceramic sealants have gained recognition in clinical practice due to their remarkable biological properties. These sealants contain components such as alumina, zirconia, bioactive glass, glass ceramics, calcium silicates, hydroxyapatite and resorbable calcium phosphates. Their properties include antimicrobial activity attributed to the formation of calcium and hydroxyl ions, resistance to moisture and blood contamination, and high biocompatibility, osteoconduction and osteogenesis facilitated by the formation of calcium hydroxide and phosphate [12,13,14]. They also exhibit reduced discolouration over time [15].

In recent years, pre-mixed bioceramic sealants have been introduced to facilitate clinical use, minimise mixing errors and improve procedural efficiency by reducing obturation time. Given the critical role of seal quality in treatment success, the use of these new formulations appears promising.

Although research on root canal obturation with bioceramic sealants has been carried out, a major limitation is that most studies have been conducted in vitro [16,17,18,19,20,21,22,23,24,25,26,27,28]. Clinical studies in patients have mainly focused on postoperative pain after root canal treatment [29,30,31,32], and the degree of healing of periapical lesions [7,32].

In this study, Neosealer Flo (Avalon Biomed, Houston, TX, USA) was selected because of its unique properties, including high cellular viability, which is associated with greater osteoconductive capacity, and high alkalinity, which allows for continuous release of calcium ions, contributing to improved apical healing [33]. Whilst there are several bioceramic sealers available on the market, Neosealer Flo stands out for these characteristics and may offer additional advantages in the resolution of periapical lesions, making it an attractive choice for this study. Furthermore, there is a lack of studies evaluating postoperative pain in RCTs obturated with this sealer.

Based on the above considerations, the objectives of our study were to evaluate the healing of periapical lesions and postoperative pain in patients undergoing RCT obturated with AH Plus and Neosealer Flo sealants. The null hypothesis was that there would be no statistically significant differences between the two sealers in their ability to induce periapical lesion healing and no differences in postoperative pain at 24 and 48 h and at 7 days.

## 2. Materials and Methods

### 2.1. Study Design

This study was designed as a single-blind, randomised clinical trial to compare the quality of obturation, sealer extrusion, postoperative pain and clinical outcomes of endodontically treated teeth using AH Plus (Dentsply Sirona, Charlotte, NC, USA) and Neosealer Flo (Avalon Biomed, Houston, TX, USA) sealants.

#### 2.1.1. Inclusion Criteria

Participants were eligible if they were over 18 years of age, cooperative and in good general health (ASA class I or II) [34]. Eligible participants had at least one necrotic tooth requiring endodontic treatment (premolars, molars, incisors, or canines) and provided informed consent for both the procedure and enrolment. In addition, the resulting RCTs had to meet the following criteria: root canal obturation within 2 mm of the radiographic apex and absence of voids on the final radiograph [35].

#### 2.1.2. Exclusion Criteria

Individuals who failed to fulfil the inclusion requirements were not considered for participation in the study, nor were those presenting teeth with open apices, coronal cracks extending into the root canal, root perforations, severe periodontal disease, or vertical root fractures.

### 2.2. Ethics Committee Approval

This study was approved by the Bioethics Committee of the Universidad Alfonso X el Sabio (reference number 2024_3/256) and registered in ISRCTN under number 42633118 (https://doi.org/10.1186/ISRCTN42633118). Data handling was carried out in accordance with Regulation (EU) 2016/679 of the European Parliament and of the Council of 27 April 2016 on the protection, processing and free movement of personal data. Participation was voluntary, informed consent was obtained, and no financial compensation was provided.

### 2.3. Calibration of Examiners and Classification Criteria

Prior to this study, two independent examiners participated in a calibration session. They evaluated 50 periapical radiographs of teeth at different stages: pre-treatment and post-treatment radiographic controls. The examiners were trained to assess obturation quality (within 2 mm of the radiographic apex, void-free) and endodontic treatment success and failure criteria according to the American Association of Endodontists (AAE) glossary [36]. A satisfactory level of calibration was achieved with a kappa index of 0.80.

The classification criteria included: Healed—teeth that are functional and asymptomatic, presenting no radiographic signs or only minimal evidence of apical radiolucency. Non-healed—teeth that are symptomatic and non-functional, with or without radiographic evidence of apical pathosis. Healing—teeth that remain functional and asymptomatic despite the presence of apical radiolucency, or symptomatic teeth, regardless of radiographic findings, and that continue to serve their intended function.

### 2.4. Clinical Procedure

The patients were treated at the Centre for Dental Innovation and Specialities of the Universidad Alfonso X el Sabio, Madrid, Spain, between March and June 2024.

Sealer allocation was randomised using the online tool random.org to ensure impartiality. Neither the operators nor the patients were aware of the assigned sealer until the obturation phase. The participants were divided into two groups: the AH group (treated with AH Plus) and the NEO group (treated with Neosealer Flo). According to the manufacturer, AH Plus consists of two pastes. Paste A: Araldite GY 250 epoxy resin, Araldite GY 285 epoxy resin, calcium tungstate, zirconium oxide, highly dispersed silicon dioxide and iron oxide pigments. Paste B contains dibenzyldiamine, amantadine, tricyclodecane-diamine, calcium tungstate, zirconium oxide, highly dispersed silicon dioxide and polydimethylsiloxane. Neosealer Flo contains tricalcium silicate, calcium aluminate, dicalcium silicate, grossite, tricalcium aluminate, tantalum oxide (tantalite), and polyethylene glycol, according to the manufacturer.

Local anaesthesia was administered using 4% articaine with 1:100,000 epinephrine (Normon, Madrid, Spain) to ensure a pain-free and stable working field for the endodontic procedure. The surgical field was isolated with a rubber dam (Hygenic, Coltène/Whaledent, Altstätten, Switzerland) to ensure absolute isolation, minimise contamination and protect the soft tissues.

After caries removal, pre-endodontic restoration with a composite (Universal Composite 3M™ Filtek™ Supreme XTE) (St. Paul, MN, USA) was performed if necessary to prevent saliva contamination or irrigant leakage into the oral cavity. The pulp chamber was carefully opened and the root canal orifices were located using a DG-16 probe, with magnification when necessary.

#### Endodontic Instrumentation

Pre-flaring was performed with the Endogal X rotary instrument (Sarria, Lugo, Spain) to the coronal two-thirds of the canal or until resistance was encountered.

The working length (WL) was determined using a K10 file (Dentsply Sirona, Ballaigues, Switzerland) and a Z Apex electronic apex locator (Zarc, Gijón, Spain) set 1 mm short of the 0.0 reading. This length was verified with a periapical radiograph.

Root canals were instrumented with Endogal files to the WL using instruments A (15.03), B (20.04) and C (25.04), ensuring that no instrument remained in the apical region for more than one second. Irrigation with 2.5% sodium hypochlorite was applied between each file using a Monoject syringe with a side-vented needle inserted 3 mm short of the WL. If dentin was observed on the last 3 mm of the instrument flutes after reaching the WL, instrumentation was continued with files E (30.06), F (40.06) or G (50.06) as required.

The final irrigation consisted of ultrasonic activation of 5 mL of sodium hypochlorite at a frequency of 45 kHz using the silver tip (0.2 mm) of the Z Activator system (Zarc, Gijón, Spain) at a working length of minus 2 mm for 30 s, followed by 5 mL of 17% EDTA solution for 30 s to remove the smear layer. The procedure was completed with a sodium hypochlorite rinse without activation.

The canals were dried with absorbent paper points and a master cone was selected to match the size of the final rotary instrument, ensuring a correct apical fit. The position of the master cone was checked radiographically.

Root canal obturation was performed using the warm vertical condensation technique with the assigned sealants. The last 4 mm of the gutta-percha cone was coated with sealer. A 200 °C heated heat carrier (Superendo Alpha, B&L, Fairfax, VA, USA) was inserted 3–4 mm short of the WL and the resulting apical plug was condensed with a Buchanan manual plugger (Kerr Dental, Bioggio, Switzerland). The coronal two-thirds of the canal was then filled by injecting thermoplastic gutta-percha (Suprendo Beta, B&L, Fairfax, VA, USA).

The pulp chamber was cleaned with an alcohol-soaked cotton pellet and the canal entrance was sealed with a flowable composite. A small cotton pellet was placed over the composite and covered with Cavit temporary material to ensure coronal sealing.

Postoperative obturation quality was assessed radiographically, with RCTs required to meet the criteria of root canal obturation within 2 mm of the radiographic apex and absence of voids on the final radiograph [35].

Pain was assessed using the Numerical Rating Scale (NRS). Preoperatively, the patients rated their pain from 0 (no pain) to 10 (worst pain imaginable). After the procedure, the patients were instructed to record their postoperative pain intensity at 24 and 48 h, including any analgesic use, with the name, dose and duration of the medication.

The patients were scheduled for a follow-up visit 7 days after endodontic treatment, at which time the final coronal restoration was placed and pain intensity was re-evaluated.

### 2.5. Clinical and Radiographic Follow-Up

The patients were recalled after 6 months for clinical and radiographic evaluation. Treatment outcomes were classified as success or failure based on the presence or absence of clinical signs such as hard or soft tissue inflammation, tenderness to palpation and/or percussion, and radiographic evidence of periapical healing. These assessments followed the AAE glossary of terms [36].

## 3. Results

The final sample consisted of 70 patients (33 males and 37 females).

Data analysis included both descriptive and inferential methods. Quantitative variables included pain recorded at 24 h, 48 h and 7 days, with normality tested using the Kolmogorov–Smirnov test. For these variables, mean, standard deviation, minimum and maximum values were calculated. The qualitative variables analysed included sex, sealer extrusion, initial lesion and lesion status at 6 months, with frequency tables showing the number of cases and corresponding percentages. All statistical analyses were performed using SPSS version 29.0.1.0.

The NEO group had lower pain levels and less variability at all three time points (24 h, 48 h, 7 days). Statistically significant differences between the groups were observed at 24 h (*p* = 0.005) and 7 days (*p* = 0.049). Table 1 and Table 2.

The effect of extruded sealer material on post-operative pain was investigated. At 24 h postoperatively, the mean pain level was higher in cases with sealer extrusion. However, at 48 h and 7 days, the pain levels were similar between patients with and without extrusion.

No extrusion was observed in 65.71% of cases, while 24 cases (34.29%) showed extrusion of material beyond the tooth. A chi-squared test for independence evaluated the association between the type of sealer used (AH or NEO) and the presence of extrusion. The chi-squared test showed no statistically significant differences (χ^2^ = 2.43, *p* = 0.119, df = 1).

The relationship between extrusion and pain scores at 24 h, 48 h and 7 days was evaluated using the Mann–Whitney U test. The analysis showed no statistically significant differences in any of the measurements.

In both groups, no significant correlation was observed between the presence of extrusion and pain levels, suggesting that extrusion alone does not significantly influence postoperative pain.

Chi-squared test was used to assess the healing ability of the sealants at six months. No statistically significant differences were found between the sealers (χ^2^ = 1.94, *p* = 0.378, df = 2).

## 4. Discussion

The success of endodontic treatment depends on achieving a precise three-dimensional obturation that ensures hermetic sealing of the root canals, prevents reinfection and promotes periapical healing [32,37]. Since early studies highlighted the importance of three-dimensional obturation [38], the combination of gutta-percha and sealer has been identified as the gold standard in this procedure [39,40]. However, with the development of new materials such as bioceramic sealants, there is a need to evaluate and compare their performance with traditional epoxy-resin-based sealants. This study focused on analysing key parameters including sealing ability, material extrusion, post-operative pain and six-month healing in necrotic pulp-treated teeth using AH Plus (epoxy resin) and Neosealer Flo (bioceramic) to provide updated evidence on the efficacy of each sealing option.

Bioceramic sealants have remarkable bioactive properties, characterised by the release of calcium ions and the establishment of a highly alkaline pH. These features are essential for promoting the development of mineralised tissue and the regeneration of periapical lesions [17,33,41]. These properties promote bacterial elimination and accelerate bone healing, particularly in periapical defects [42]. Compared to epoxy based sealers such as AH Plus, bioceramic sealants offer potential advantages due to their ability to induce hydroxyapatite formation and maintain a prolonged alkaline pH [43].

Recent studies [17,33] have shown that NeoSealer Flo outperforms AH Plus in terms of cellular viability and osteoconduction. Sebastián et al. [33] reported that the sustained release of calcium ions and the alkaline environment created by NeoSealer Flo not only favour periapical repair but also optimise the conditions for mineralising cells such as osteoblasts, making it a promising option for the treatment of periapical lesions.

In addition, a recent in vivo study [44] showed that NeoSealer Flo promotes the expression of interleukin-10 (IL-10), an anti-inflammatory cytokine, particularly in the peri-material regions. This response suggests that the calcium silicate in its formulation may contribute to modulating the inflammatory process, supporting a controlled immune response and creating a more favourable environment for periapical healing. These findings support the biocompatibility of NeoSealer Flo and its potential advantages in endodontic applications.

In addition, NeoSealer Flo demonstrated advantages in obturation techniques such as reduced void formation and improved adaptability in endodontic sealing. Kim et al. [45] documented a void volume of 1.8% and reduced coronal porosity when using warm vertical condensation techniques. Thus, the efficacy of NeoSealer Flo is enhanced by the quality of the obturation techniques used. In particular, vertical condensation has been widely recognised for its ability to provide an effective three-dimensional seal while minimising gaps that could harbour bacteria, thereby reducing the risk of treatment failure [46,47].

Pain following RCTs is a frequently reported complication [48,49,50,51], with prevalence rates ranging from 2% to 83%, making it one of the most common adverse effects of RCT [52,53]. This phenomenon is multifactorial, influenced by patient intrinsic factors, tooth characteristics, pre-existing pathology, the materials used and the procedure itself [54,55].

Several studies [29,56,57,58] have analysed the postoperative pain associated with different root canal sealants, although the results are heterogeneous. Some reports [29,56,57,59,60] suggest that epoxy-resin-based sealants are not associated with a higher incidence of postoperative pain than bioceramic sealants, indicating that the differences between the two types are not significant.

However, other studies [61,62] have found that calcium-silicate-based sealers are associated with significantly lower pain levels than epoxy-resin-based sealers (AH Plus) within the first 6 to 24 h. Beyond this period, at 48 h and on days 5 and 7, no significant differences in postoperative pain were observed. These observations are consistent with our results showing a significant reduction in postoperative pain during the first week.

In our investigation, greater pain intensity was associated with cases of sealer extrusion in both groups, attributed to mechanical irritation caused by over-instrumentation of the apical foramen or by extruded material in the periapical tissues. Sathor et al. [52] suggested that extrusion of endodontic sealant through the apical foramen or a lateral canal may cause inflammation, tissue degeneration and postoperative pain.

Regarding healing capacity, both sealers were evaluated at the six-month follow-up using clinical and radiographic criteria. The comparison between the AH and NEO groups showed no statistically significant differences (*p* = 0.378), suggesting comparable efficacy in facilitating the resolution of periapical lesions. These results are consistent with previous reports in the literature [33,45], which have highlighted the comparable efficacy of bioceramic and epoxy sealants in the healing process. In terms of healing, bioceramic sealants offer theoretical advantages such as calcium ion release, highly alkaline pH and hydroxyapatite formation, which promote bone regeneration and tissue repair [41,42,43]. However, although the trend in this study appeared favourable for the NEO group, it did not translate into clinically significant differences.

The instrumentation and irrigation phases are critical to the overall success of root canal treatment. In the present study, sodium hypochlorite was used for irrigation between each file, and final activation with the Z Activator was performed to remove the smear layer created during canal preparation [63]. Removal of this smear layer enhances the antimicrobial effect of sodium hypochlorite by exposing residual bacteria, thereby facilitating more effective obturation and contributing to better conditions for periapical healing [64]. Eliminating this layer enhances the antimicrobial action of sodium hypochlorite by exposing residual bacteria, thereby facilitating a more effective obturation and contributing to better conditions for periapical healing.

Finally, while the AAE glossary of terms has been used to assess clinical outcomes [36], some studies have used the periapical index (PAI) [65,66,67,68]. However, the PAI has limitations, particularly in teeth with multiple roots or variations in bone morphology and density, which may introduce bias [69]. The AAE terminology is more appropriate for clinical practice as it allows for a comprehensive interpretation of clinical signs and symptoms in addition to radiographic images, allowing for a clear classification of cases into healed, healing or failed categories.

Our results suggest that NeoSealer Flo is a promising option for reducing post-operative pain, particularly within the first 24 h, without compromising seal quality or healing capacity. While AH Plus remains a widely accepted standard, bioceramic sealants such as NeoSealer Flo offer advantages such as ease of use and bioactive properties, positioning them as an attractive alternative in endodontic therapy.

## 5. Conclusions

NeoSealer Flo was associated with a lower incidence of postoperative pain at 24 h and 7 days compared to AH Plus. Material extrusion did not show a significant effect on pain or a clear correlation with the type of sealer used. Both materials achieved similar periapical healing rates. NeoSealer Flo showed advantages in pain reduction, while both sealers showed comparable efficacy in terms of healing.

## 6. Future Research Directions

Future studies should focus on volumetric analysis of healing using CBCT. In addition, comparative in vivo studies of the interaction of bioceramic sealers with periapical tissues could provide valuable insights into their regenerative potential and clinical impact at the tissue level.

## Figures and Tables

**Table 1:** Kolmogorov–Smirnov normality test.

Sealer	Variable	N	Min	Max	Mean	SD	*p*-Value
AH	Pain 24 h	36	0	10	0.97	2.27	<0.001
	Pain 48 h			8	0.53	1.61	<0.001
	Pain 7 days			3	0.17	0.56	<0.001
NEO	Pain 24 h	34	0	2	0.06	0.34	<0.001
	Pain 48 h			1	0.03	0.17	<0.001
	Pain 7 days			0	0.0	0.0	<0.001

**Table 2:** Mann–Whitney U test for pain.

Variable	Statistic	*p*-Value
Pain 24 h	764.0	0.005 ** (AH > NEO)
Pain 48 h	697.5	0.055
Pain 7 days	680.0	0.049 * (AH > NEO)

* *p*-value < 0.05—statistically significant, ** *p*-value < 0.01—high statistically significant.

## Data Availability

The data that support the findings of this study are available from the corresponding author upon reasonable request.
